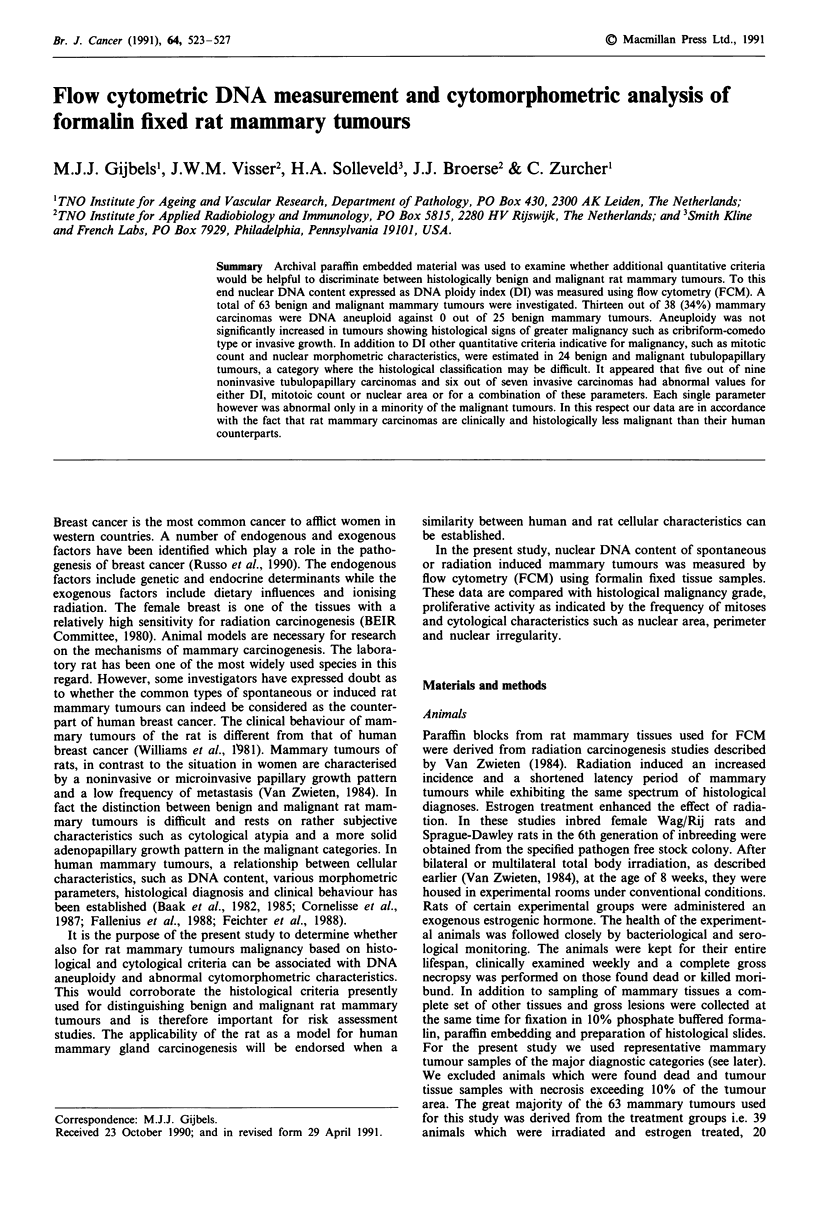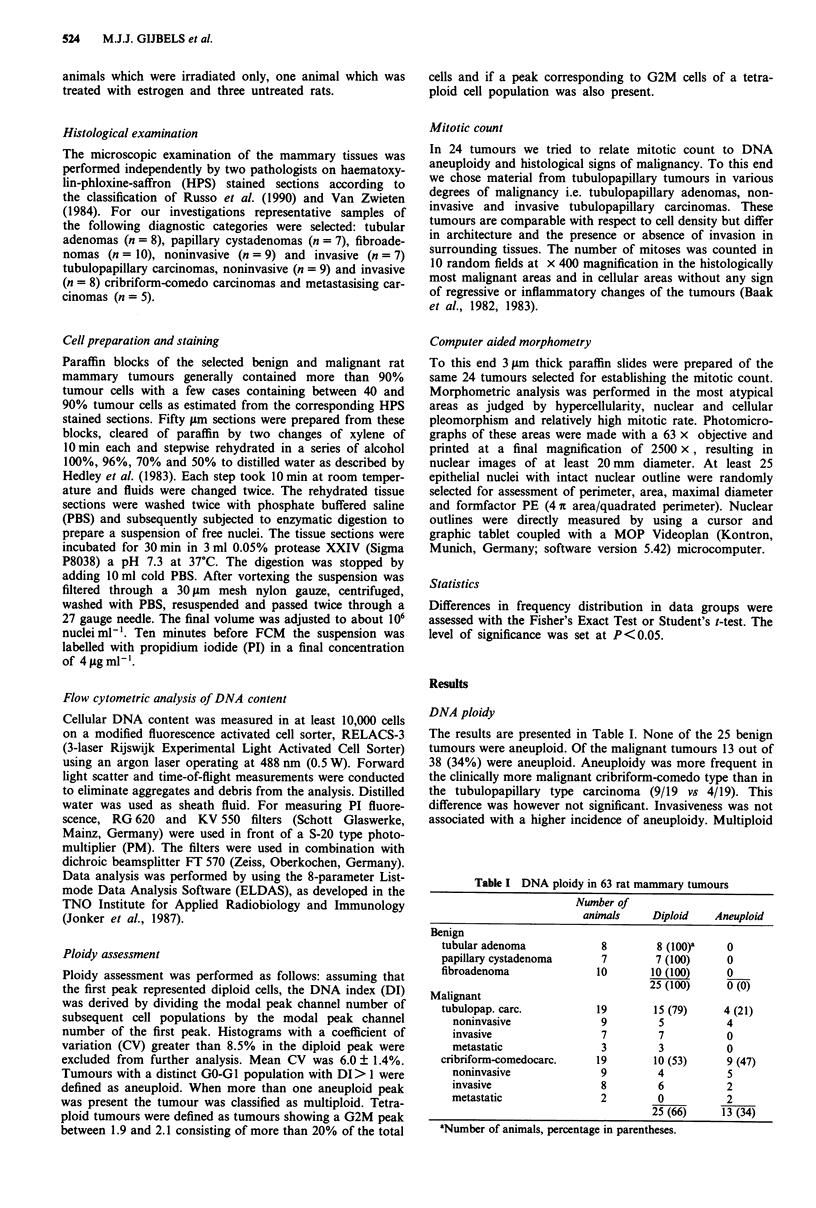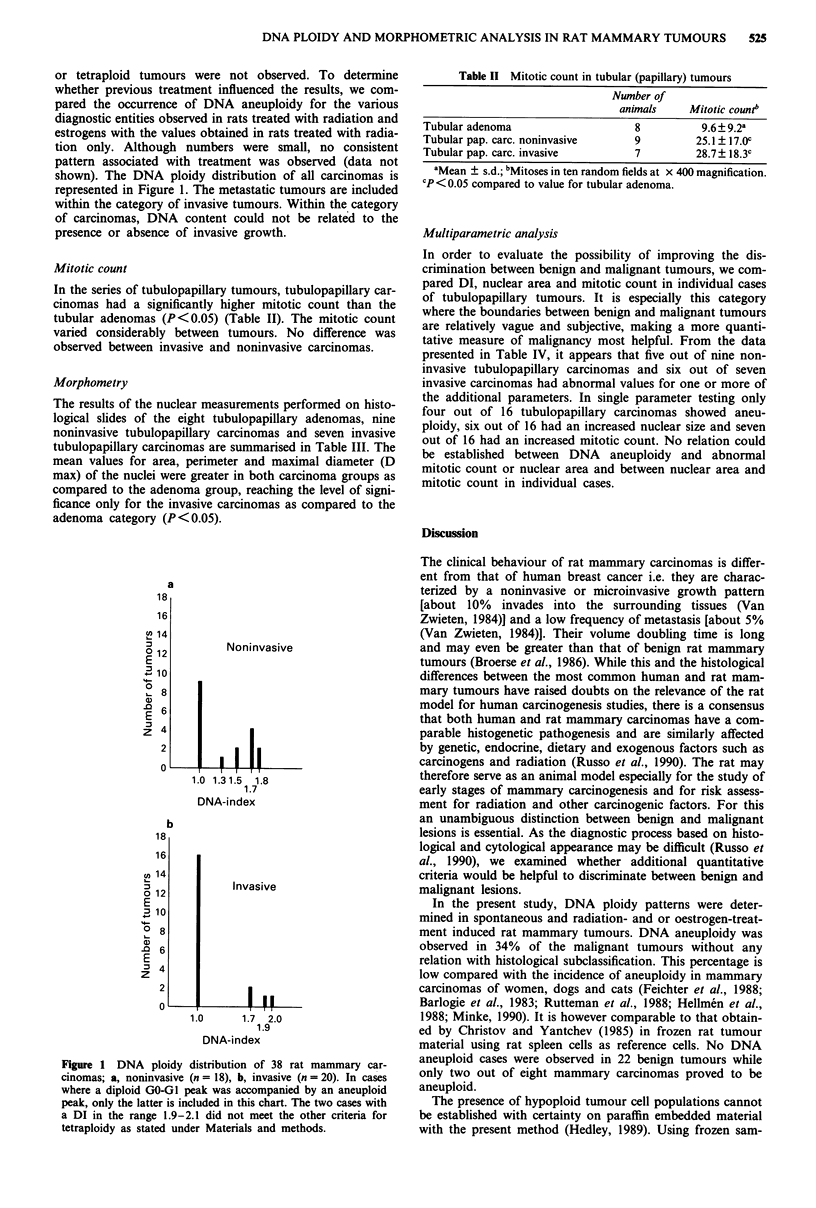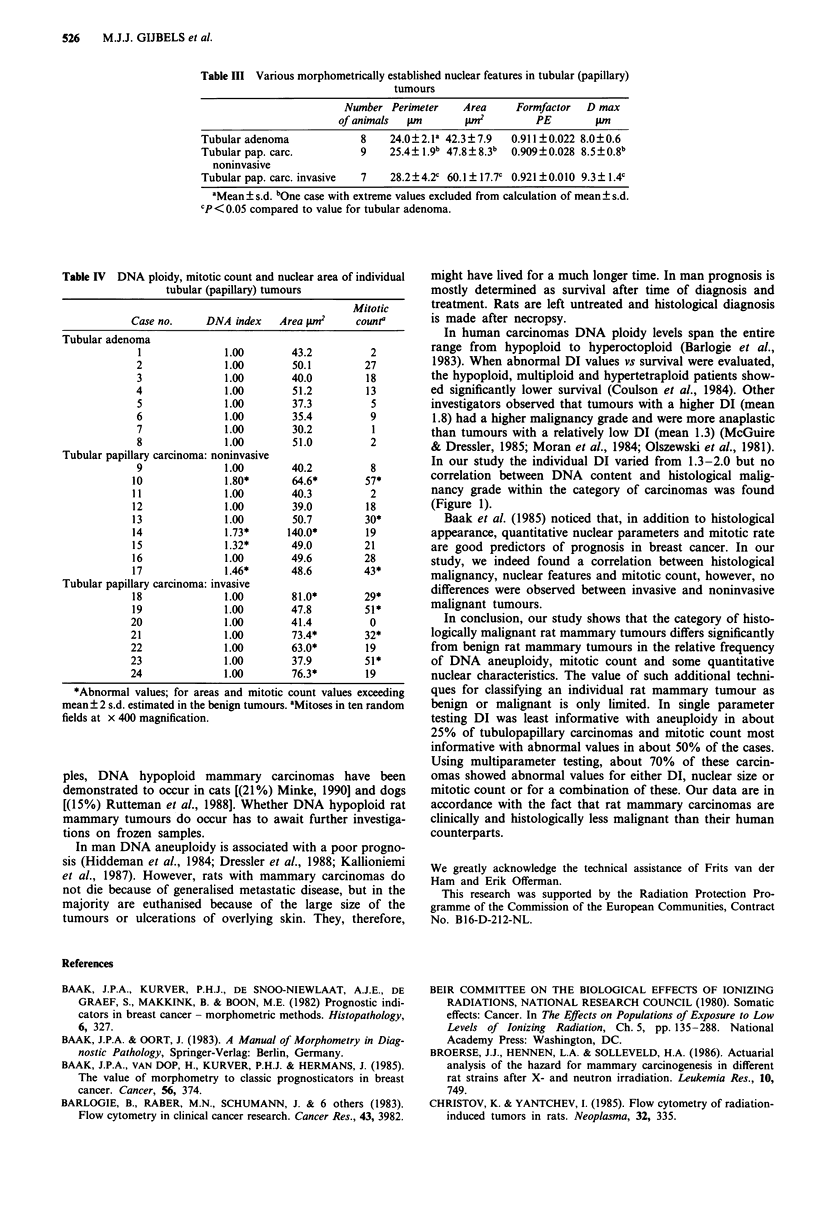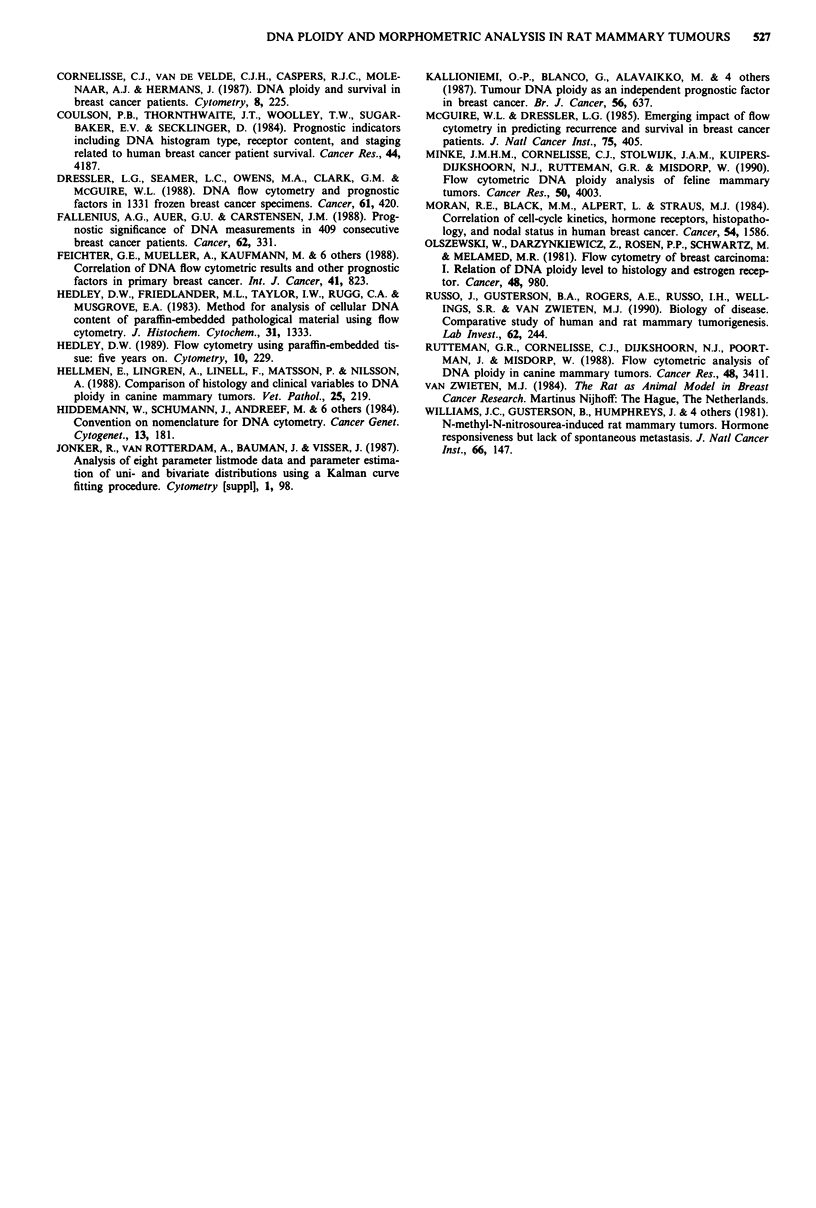# Flow cytometric DNA measurement and cytomorphometric analysis of formalin fixed rat mammary tumours.

**DOI:** 10.1038/bjc.1991.342

**Published:** 1991-09

**Authors:** M. J. Gijbels, J. W. Visser, H. A. Solleveld, J. J. Broerse, C. Zurcher

**Affiliations:** TNO Institute for Ageing and Vascular Research, Department of Pathology, Leiden, The Netherlands.

## Abstract

Archival paraffin embedded material was used to examine whether additional quantitative criteria would be helpful to discriminate between histologically benign and malignant rat mammary tumours. To this end nuclear DNA content expressed as DNA ploidy index (DI) was measured using flow cytometry (FCM). A total of 63 benign and malignant mammary tumours were investigated. Thirteen out of 38 (34%) mammary carcinomas were DNA aneuploid against 0 out of 25 benign mammary tumours. Aneuploidy was not significantly increased in tumours showing histological signs of greater malignancy such as cribriform-comedo type or invasive growth. In addition to DI other quantitative criteria indicative for malignancy, such as mitotic count and nuclear morphometric characteristics, were estimated in 24 benign and malignant tubulopapillary tumours, a category where the histological classification may be difficult. It appeared that five out of nine noninvasive tubulopapillary carcinomas and six out of seven invasive carcinomas had abnormal values for either DI, mitotic count or nuclear area or for a combination of these parameters. Each single parameter however was abnormal only in a minority of the malignant tumours. In this respect our data are in accordance with the fact that rat mammary carcinomas are clinically and histologically less malignant than their human counterparts.


*Br. J. Cancer* (1991), **64**, 523–527 © Macmillan Press Ltd., 1991

### Flow cytometric DNA measurement and cytomorphometric analysis of formalin fixed rat mammary tumours

M.J.J. Gijbels[1], J.W.M. Visser[2], H.A. Solleveld[3], J.J. Broerse[2] & C. Zurcher[1]

[1]*TNO Institute for Ageing and Vascular Research, Department of Pathology, PO Box 430, 2300 AK Leiden, The Netherlands;* [2]*TNO Institute for Applied Radiobiology and Immunology, PO Box 5815, 2280 HV Rijswijk, The Netherlands; and* [3]*Smith Kline and French Labs, PO Box 7929, Philadelphia, Pennsylvania 19101, USA.*

**Summary** Archival paraffin embedded material was used to examine whether additional quantitative criteria would be helpful to discriminate between histologically benign and malignant rat mammary tumours. To this end nuclear DNA content expressed as DNA ploidy index (DI) was measured using flow cytometry (FCM). A total of 63 benign and malignant mammary tumours were investigated. Thirteen out of 38 (34%) mammary carcinomas were DNA aneuploid against 0 out of 25 benign mammary tumours. Aneuploidy was not significantly increased in tumours showing histological signs of greater malignancy such as cribriform-comedo type or invasive growth. In addition to DI other quantitative criteria indicative for malignancy, such as mitotic count and nuclear morphometric characteristics, were estimated in 24 benign and malignant tubulopapillary tumours, a category where the histological classification may be difficult. It appeared that five out of nine noninvasive tubulopapillary carcinomas and six out of seven invasive carcinomas had abnormal values for either DI, mitotoic count or nuclear area or for a combination of these parameters. Each single parameter however was abnormal only in a minority of the malignant tumours. In this respect our data are in accordance with the fact that rat mammary carcinomas are clinically and histologically less malignant than their human counterparts.

Breast cancer is the most common cancer to afflict women in western countries. A number of endogenous and exogenous factors have been identified which play a role in the pathogenesis of breast cancer (Russo *et al.*, 1990). The endogenous factors include genetic and endocrine determinants while the exogenous factors include dietary influences and ionising radiation. The female breast is one of the tissues with a relatively high sensitivity for radiation carcinogenesis (BEIR Committee, 1980). Animal models are necessary for research on the mechanisms of mammary carcinogenesis. The laboratory rat has been one of the most widely used species in this regard. However, some investigators have expressed doubt as to whether the common types of spontaneous or induced rat mammary tumours can indeed be considered as the counterpart of human breast cancer. The clinical behaviour of mammary tumours of the rat is different from that of human breast cancer (Williams *et al.*, 1981). Mammary tumours of rats, in contrast to the situation in women are characterised by a noninvasive or microinvasive papillary growth pattern and a low frequency of metastasis (Van Zwieten, 1984). In fact the distinction between benign and malignant rat mammary tumours is difficult and rests on rather subjective characteristics such as cytological atypia and a more solid adenopapillary growth pattern in the malignant categories. In human mammary tumours, a relationship between cellular characteristics, such as DNA content, various morphometric parameters, histological diagnosis and clinical behaviour has been established (Baak *et al.*, 1982, 1985; Cornelisse *et al.*, 1987; Fallenius *et al.*, 1988; Feichter *et al.*, 1988).

It is the purpose of the present study to determine whether also for rat mammary tumours malignancy based on histological and cytological criteria can be associated with DNA aneuploidy and abnormal cytomorphometric characteristics. This would corroborate the histological criteria presently used for distinguishing benign and malignant rat mammary tumours and is therefore important for risk assessment studies. The applicability of the rat as a model for human mammary gland carcinogenesis will be endorsed when a similarity between human and rat cellular characteristics can be established.

In the present study, nuclear DNA content of spontaneous or radiation induced mammary tumours was measured by flow cytometry (FCM) using formalin fixed tissue samples. These data are compared with histological malignancy grade, proliferative activity as indicated by the frequency of mitoses and cytological characteristics such as nuclear area, perimeter and nuclear irregularity.

### Materials and methods

### *Animals*

Paraffin blocks from rat mammary tissues used for FCM were derived from radiation carcinogenesis studies described by Van Zwieten (1984). Radiation induced an increased incidence and a shortened latency period of mammary tumours while exhibiting the same spectrum of histological diagnoses. Estrogen treatment enhanced the effect of radiation. In these studies inbred female Wag/Rij rats and Sprague-Dawley rats in the 6th generation of inbreeding were obtained from the specified pathogen free stock colony. After bilateral or multilateral total body irradiation, as described earlier (Van Zwieten, 1984), at the age of 8 weeks, they were housed in experimental rooms under conventional conditions. Rats of certain experimental groups were administered an exogenous estrogenic hormone. The health of the experimental animals was followed closely by bacteriological and serological monitoring. The animals were kept for their entire lifespan, clinically examined weekly and a complete gross necropsy was performed on those found dead or killed moribund. In addition to sampling of mammary tissues a complete set of other tissues and gross lesions were collected at the same time for fixation in 10% phosphate buffered formalin, paraffin embedding and preparation of histological slides. For the present study we used representative mammary tumour samples of the major diagnostic categories (see later). We excluded animals which were found dead and tumour tissue samples with necrosis exceeding 10% of the tumour area. The great majority of the 63 mammary tumours used for this study was derived from the treatment groups i.e. 39 animals which were irradiated and estrogen treated, 20

Correspondence: M.J.J. Gijbels.
Received 23 October 1990; and in revised form 29 April 1991.

animals which were irradiated only, one animal which was treated with estrogen and three untreated rats.

*Histological examination*

The microscopic examination of the mammary tissues was performed independently by two pathologists on haematoxylin-phloxine-saffron (HPS) stained sections according to the classification of Russo *et al.* (1990) and Van Zwieten (1984). For our investigations representative samples of the following diagnostic categories were selected: tubular adenomas ($n = 8$), papillary cystadenomas ($n = 7$), fibroadenomas ($n = 10$), noninvasive ($n = 9$) and invasive ($n = 7$) tubulopapillary carcinomas, noninvasive ($n = 9$) and invasive ($n = 8$) cribriform-comedo carcinomas and metastasising carcinomas ($n = 5$).

*Cell preparation and staining*

Paraffin blocks of the selected benign and malignant rat mammary tumours generally contained more than 90% tumour cells with a few cases containing between 40 and 90% tumour cells as estimated from the corresponding HPS stained sections. Fifty μm sections were prepared from these blocks, cleared of paraffin by two changes of xylene of 10 min each and stepwise rehydrated in a series of alcohol 100%, 96%, 70% and 50% to distilled water as described by Hedley *et al.* (1983). Each step took 10 min at room temperature and fluids were changed twice. The rehydrated tissue sections were washed twice with phosphate buffered saline (PBS) and subsequently subjected to enzymatic digestion to prepare a suspension of free nuclei. The tissue sections were incubated for 30 min in 3 ml 0.05% protease XXIV (Sigma P8038) a pH 7.3 at 37°C. The digestion was stopped by adding 10 ml cold PBS. After vortexing the suspension was filtered through a 30 μm mesh nylon gauze, centrifuged, washed with PBS, resuspended and passed twice through a 27 gauge needle. The final volume was adjusted to about $10^6$ nuclei $ml^{-1}$. Ten minutes before FCM the suspension was labelled with propidium iodide (PI) in a final concentration of 4 $\mu g\ ml^{-1}$.

*Flow cytometric analysis of DNA content*

Cellular DNA content was measured in at least 10,000 cells on a modified fluorescence activated cell sorter, RELACS-3 (3-laser Rijswijk Experimental Light Activated Cell Sorter) using an argon laser operating at 488 nm (0.5 W). Forward light scatter and time-of-flight measurements were conducted to eliminate aggregates and debris from the analysis. Distilled water was used as sheath fluid. For measuring PI fluorescence, RG 620 and KV 550 filters (Schott Glaswerke, Mainz, Germany) were used in front of a S-20 type photomultiplier (PM). The filters were used in combination with dichroic beamsplitter FT 570 (Zeiss, Oberkochen, Germany). Data analysis was performed by using the 8-parameter Listmode Data Analysis Software (ELDAS), as developed in the TNO Institute for Applied Radiobiology and Immunology (Jonker *et al.*, 1987).

*Ploidy assessment*

Ploidy assessment was performed as follows: assuming that the first peak represented diploid cells, the DNA index (DI) was derived by dividing the modal peak channel number of subsequent cell populations by the modal peak channel number of the first peak. Histograms with a coefficient of variation (CV) greater than 8.5% in the diploid peak were excluded from further analysis. Mean CV was 6.0 ± 1.4%. Tumours with a distinct G0-G1 population with DI > 1 were defined as aneuploid. When more than one aneuploid peak was present the tumour was classified as multiploid. Tetraploid tumours were defined as tumours showing a G2M peak between 1.9 and 2.1 consisting of more than 20% of the total cells and if a peak corresponding to G2M cells of a tetraploid cell population was also present.

*Mitotic count*

In 24 tumours we tried to relate mitotic count to DNA aneuploidy and histological signs of malignancy. To this end we chose material from tubulopapillary tumours in various degrees of malignancy i.e. tubulopapillary adenomas, noninvasive and invasive tubulopapillary carcinomas. These tumours are comparable with respect to cell density but differ in architecture and the presence or absence of invasion in surrounding tissues. The number of mitoses was counted in 10 random fields at × 400 magnification in the histologically most malignant areas and in cellular areas without any sign of regressive or inflammatory changes of the tumours (Baak *et al.*, 1982, 1983).

*Computer aided morphometry*

To this end 3 μm thick paraffin slides were prepared of the same 24 tumours selected for establishing the mitotic count. Morphometric analysis was performed in the most atypical areas as judged by hypercellularity, nuclear and cellular pleomorphism and relatively high mitotic rate. Photomicrographs of these areas were made with a 63 × objective and printed at a final magnification of 2500 ×, resulting in nuclear images of at least 20 mm diameter. At least 25 epithelial nuclei with intact nuclear outline were randomly selected for assessment of perimeter, area, maximal diameter and formfactor PE (4 π area/quadrated perimeter). Nuclear outlines were directly measured by using a cursor and graphic tablet coupled with a MOP Videoplan (Kontron, Munich, Germany; software version 5.42) microcomputer.

*Statistics*

Differences in frequency distribution in data groups were assessed with the Fisher's Exact Test or Student's *t*-test. The level of significance was set at $P < 0.05$.

### Results

*DNA ploidy*

The results are presented in Table I. None of the 25 benign tumours were aneuploid. Of the malignant tumours 13 out of 38 (34%) were aneuploid. Aneuploidy was more frequent in the clinically more malignant cribriform-comedo type than in the tubulopapillary type carcinoma (9/19 *vs* 4/19). This difference was however not significant. Invasiveness was not associated with a higher incidence of aneuploidy. Multiploid

**Table I** DNA ploidy in 63 rat mammary tumours

| | *Number of animals* | *Diploid* | *Aneuploid* |
|---|---|---|---|
| Benign | | | |
| tubular adenoma | 8 | 8 (100)[a] | 0 |
| papillary cystadenoma | 7 | 7 (100) | 0 |
| fibroadenoma | 10 | 10 (100) | 0 |
| | | 25 (100) | 0 (0) |
| Malignant | | | |
| tubulopap. carc. | 19 | 15 (79) | 4 (21) |
| noninvasive | 9 | 5 | 4 |
| invasive | 7 | 7 | 0 |
| metastatic | 3 | 3 | 0 |
| cribriform-comedocarc. | 19 | 10 (53) | 9 (47) |
| noninvasive | 9 | 4 | 5 |
| invasive | 8 | 6 | 2 |
| metastatic | 2 | 0 | 2 |
| | | 25 (66) | 13 (34) |

[a]Number of animals, percentage in parentheses.

or tetraploid tumours were not observed. To determine whether previous treatment influenced the results, we compared the occurrence of DNA aneuploidy for the various diagnostic entities observed in rats treated with radiation and estrogens with the values obtained in rats treated with radiation only. Although numbers were small, no consistent pattern associated with treatment was observed (data not shown). The DNA ploidy distribution of all carcinomas is represented in Figure 1. The metastatic tumours are included within the category of invasive tumours. Within the category of carcinomas, DNA content could not be related to the presence or absence of invasive growth.

*Mitotic count*

In the series of tubulopapillary tumours, tubulopapillary carcinomas had a significantly higher mitotic count than the tubular adenomas ($P<0.05$) (Table II). The mitotic count varied considerably between tumours. No difference was observed between invasive and noninvasive carcinomas.

*Morphometry*

The results of the nuclear measurements performed on histological slides of the eight tubulopapillary adenomas, nine noninvasive tubulopapillary carcinomas and seven invasive tubulopapillary carcinomas are summarised in Table III. The mean values for area, perimeter and maximal diameter (D max) of the nuclei were greater in both carcinoma groups as compared to the adenoma group, reaching the level of significance only for the invasive carcinomas as compared to the adenoma category ($P<0.05$).

**Figure 1** DNA ploidy distribution of 38 rat mammary carcinomas; **a**, noninvasive ($n=18$), **b**, invasive ($n=20$). In cases where a diploid G0-G1 peak was accompanied by an aneuploid peak, only the latter is included in this chart. The two cases with a DI in the range 1.9–2.1 did not meet the other criteria for tetraploidy as stated under Materials and methods.

**Table II** Mitotic count in tubular (papillary) tumours

| | *Number of animals* | *Mitotic count*[b] |
|---|---|---|
| Tubular adenoma | 8 | 9.6±9.2[a] |
| Tubular pap. carc. noninvasive | 9 | 25.1±17.0[c] |
| Tubular pap. carc. invasive | 7 | 28.7±18.3[c] |

[a]Mean ± s.d.; [b]Mitoses in ten random fields at × 400 magnification. [c]$P<0.05$ compared to value for tubular adenoma.

*Multiparametric analysis*

In order to evaluate the possibility of improving the discrimination between benign and malignant tumours, we compared DI, nuclear area and mitotic count in individual cases of tubulopapillary tumours. It is especially this category where the boundaries between benign and malignant tumours are relatively vague and subjective, making a more quantitative measure of malignancy most helpful. From the data presented in Table IV, it appears that five out of nine noninvasive tubulopapillary carcinomas and six out of seven invasive carcinomas had abnormal values for one or more of the additional parameters. In single parameter testing only four out of 16 tubulopapillary carcinomas showed aneuploidy, six out of 16 had an increased nuclear size and seven out of 16 had an increased mitotic count. No relation could be established between DNA aneuploidy and abnormal mitotic count or nuclear area and between nuclear area and mitotic count in individual cases.

### Discussion

The clinical behaviour of rat mammary carcinomas is different from that of human breast cancer i.e. they are characterized by a noninvasive or microinvasive growth pattern [about 10% invades into the surrounding tissues (Van Zwieten, 1984)] and a low frequency of metastasis [about 5% (Van Zwieten, 1984)]. Their volume doubling time is long and may even be greater than that of benign rat mammary tumours (Broerse *et al.*, 1986). While this and the histological differences between the most common human and rat mammary tumours have raised doubts on the relevance of the rat model for human carcinogenesis studies, there is a consensus that both human and rat mammary carcinomas have a comparable histogenetic pathogenesis and are similarly affected by genetic, endocrine, dietary and exogenous factors such as carcinogens and radiation (Russo *et al.*, 1990). The rat may therefore serve as an animal model especially for the study of early stages of mammary carcinogenesis and for risk assessment for radiation and other carcinogenic factors. For this an unambiguous distinction between benign and malignant lesions is essential. As the diagnostic process based on histological and cytological appearance may be difficult (Russo *et al.*, 1990), we examined whether additional quantitative criteria would be helpful to discriminate between benign and malignant lesions.

In the present study, DNA ploidy patterns were determined in spontaneous and radiation- and or oestrogen-treatment induced rat mammary tumours. DNA aneuploidy was observed in 34% of the malignant tumours without any relation with histological subclassification. This percentage is low compared with the incidence of aneuploidy in mammary carcinomas of women, dogs and cats (Feichter *et al.*, 1988; Barlogie *et al.*, 1983; Rutteman *et al.*, 1988; Hellmén *et al.*, 1988; Minke, 1990). It is however comparable to that obtained by Christov and Yantchev (1985) in frozen rat tumour material using rat spleen cells as reference cells. No DNA aneuploid cases were observed in 22 benign tumours while only two out of eight mammary carcinomas proved to be aneuploid.

The presence of hypoploid tumour cell populations cannot be established with certainty on paraffin embedded material with the present method (Hedley, 1989). Using frozen sam-

**Table III** Various morphometrically established nuclear features in tubular (papillary) tumours

| | *Number of animals* | *Perimeter μm* | *Area μm²* | *Formfactor PE* | *D max μm* |
|---|---|---|---|---|---|
| Tubular adenoma | 8 | 24.0±2.1[a] | 42.3±7.9 | 0.911±0.022 | 8.0±0.6 |
| Tubular pap. carc. noninvasive | 9 | 25.4±1.9[b] | 47.8±8.3[b] | 0.909±0.028 | 8.5±0.8[b] |
| Tubular pap. carc. invasive | 7 | 28.2±4.2[c] | 60.1±17.7[c] | 0.921±0.010 | 9.3±1.4[c] |

[a]Mean±s.d. [b]One case with extreme values excluded from calculation of mean±s.d. [c]$P<0.05$ compared to value for tubular adenoma.

**Table IV** DNA ploidy, mitotic count and nuclear area of individual tubular (papillary) tumours

| *Case no.* | *DNA index* | *Area μm²* | *Mitotic count[a]* |
|---|---|---|---|
| Tubular adenoma | | | |
| 1 | 1.00 | 43.2 | 2 |
| 2 | 1.00 | 50.1 | 27 |
| 3 | 1.00 | 40.0 | 18 |
| 4 | 1.00 | 51.2 | 13 |
| 5 | 1.00 | 37.3 | 5 |
| 6 | 1.00 | 35.4 | 9 |
| 7 | 1.00 | 30.2 | 1 |
| 8 | 1.00 | 51.0 | 2 |
| Tubular papillary carcinoma: noninvasive | | | |
| 9 | 1.00 | 40.2 | 8 |
| 10 | 1.80* | 64.6* | 57* |
| 11 | 1.00 | 40.3 | 2 |
| 12 | 1.00 | 39.0 | 18 |
| 13 | 1.00 | 50.7 | 30* |
| 14 | 1.73* | 140.0* | 19 |
| 15 | 1.32* | 49.0 | 21 |
| 16 | 1.00 | 49.6 | 28 |
| 17 | 1.46* | 48.6 | 43* |
| Tubular papillary carcinoma: invasive | | | |
| 18 | 1.00 | 81.0* | 29* |
| 19 | 1.00 | 47.8 | 51* |
| 20 | 1.00 | 41.4 | 0 |
| 21 | 1.00 | 73.4* | 32* |
| 22 | 1.00 | 63.0* | 19 |
| 23 | 1.00 | 37.9 | 51* |
| 24 | 1.00 | 76.3* | 19 |

*Abnormal values; for areas and mitotic count values exceeding mean±2 s.d. estimated in the benign tumours. [a]Mitoses in ten random fields at × 400 magnification.

ples, DNA hypoploid mammary carcinomas have been demonstrated to occur in cats [(21%) Minke, 1990] and dogs [(15%) Rutteman *et al.*, 1988]. Whether DNA hypoploid rat mammary tumours do occur has to await further investigations on frozen samples.

In man DNA aneuploidy is associated with a poor prognosis (Hiddeman *et al.*, 1984; Dressler *et al.*, 1988; Kallioniemi *et al.*, 1987). However, rats with mammary carcinomas do not die because of generalised metastatic disease, but in the majority are euthanised because of the large size of the tumours or ulcerations of overlying skin. They, therefore, might have lived for a much longer time. In man prognosis is mostly determined as survival after time of diagnosis and treatment. Rats are left untreated and histological diagnosis is made after necropsy.

In human carcinomas DNA ploidy levels span the entire range from hypoploid to hyperoctoploid (Barlogie *et al.*, 1983). When abnormal DI values *vs* survival were evaluated, the hypoploid, multiploid and hypertetraploid patients showed significantly lower survival (Coulson *et al.*, 1984). Other investigators observed that tumours with a higher DI (mean 1.8) had a higher malignancy grade and were more anaplastic than tumours with a relatively low DI (mean 1.3) (McGuire & Dressler, 1985; Moran *et al.*, 1984; Olszewski *et al.*, 1981). In our study the individual DI varied from 1.3–2.0 but no correlation between DNA content and histological malignancy grade within the category of carcinomas was found (Figure 1).

Baak *et al.* (1985) noticed that, in addition to histological appearance, quantitative nuclear parameters and mitotic rate are good predictors of prognosis in breast cancer. In our study, we indeed found a correlation between histological malignancy, nuclear features and mitotic count, however, no differences were observed between invasive and noninvasive malignant tumours.

In conclusion, our study shows that the category of histologically malignant rat mammary tumours differs significantly from benign rat mammary tumours in the relative frequency of DNA aneuploidy, mitotic count and some quantitative nuclear characteristics. The value of such additional techniques for classifying an individual rat mammary tumour as benign or malignant is only limited. In single parameter testing DI was least informative with aneuploidy in about 25% of tubulopapillary carcinomas and mitotic count most informative with abnormal values in about 50% of the cases. Using multiparameter testing, about 70% of these carcinomas showed abnormal values for either DI, nuclear size or mitotic count or for a combination of these. Our data are in accordance with the fact that rat mammary carcinomas are clinically and histologically less malignant than their human counterparts.

We greatly acknowledge the technical assistance of Frits van der Ham and Erik Offerman.

This research was supported by the Radiation Protection Programme of the Commission of the European Communities, Contract No. B16-D-212-NL.

### References

BAAK, J.P.A., KURVER, P.H.J., DE SNOO-NIEWLAAT, A.J.E., DE GRAEF, S., MAKKINK, B. & BOON, M.E. (1982) Prognostic indicators in breast cancer – morphometric methods. *Histopathology*, **6**, 327.

BAAK, J.P.A. & OORT, J. (1983). *A Manual of Morphometry in Diagnostic Pathology*, Springer-Verlag: Berlin, Germany.

BAAK, J.P.A., VAN DOP, H., KURVER, P.H.J. & HERMANS, J. (1985). The value of morphometry to classic prognosticators in breast cancer. *Cancer*, **56**, 374.

BARLOGIE, B., RABER, M.N., SCHUMANN, J. & 6 others (1983). Flow cytometry in clinical cancer research. *Cancer Res.*, **43**, 3982.

BEIR COMMITTEE ON THE BIOLOGICAL EFFECTS OF IONIZING RADIATIONS, NATIONAL RESEARCH COUNCIL (1980). Somatic effects: Cancer. In *The Effects on Populations of Exposure to Low Levels of Ionizing Radiation*, Ch. 5, pp. 135–288. National Academy Press: Washington, DC.

BROERSE, J.J., HENNEN, L.A. & SOLLEVELD, H.A. (1986). Actuarial analysis of the hazard for mammary carcinogenesis in different rat strains after X- and neutron irradiation. *Leukemia Res.*, **10**, 749.

CHRISTOV, K. & YANTCHEV, I. (1985). Flow cytometry of radiation-induced tumors in rats. *Neoplasma*, **32**, 335.

CORNELISSE, C.J., VAN DE VELDE, C.J.H., CASPERS, R.J.C., MOLENAAR, A.J. & HERMANS, J. (1987). DNA ploidy and survival in breast cancer patients. *Cytometry*, **8**, 225.

COULSON, P.B., THORNTHWAITE, J.T., WOOLLEY, T.W., SUGARBAKER, E.V. & SECKLINGER, D. (1984). Prognostic indicators including DNA histogram type, receptor content, and staging related to human breast cancer patient survival. *Cancer Res.*, **44**, 4187.

DRESSLER, L.G., SEAMER, L.C., OWENS, M.A., CLARK, G.M. & MCGUIRE, W.L. (1988). DNA flow cytometry and prognostic factors in 1331 frozen breast cancer specimens. *Cancer*, **61**, 420.

FALLENIUS, A.G., AUER, G.U. & CARSTENSEN, J.M. (1988). Prognostic significance of DNA measurements in 409 consecutive breast cancer patients. *Cancer*, **62**, 331.

FEICHTER, G.E., MUELLER, A., KAUFMANN, M. & 6 others (1988). Correlation of DNA flow cytometric results and other prognostic factors in primary breast cancer. *Int. J. Cancer*, **41**, 823.

HEDLEY, D.W., FRIEDLANDER, M.L., TAYLOR, I.W., RUGG, C.A. & MUSGROVE, E.A. (1983). Method for analysis of cellular DNA content of paraffin-embedded pathological material using flow cytometry. *J. Histochem. Cytochem.*, **31**, 1333.

HEDLEY, D.W. (1989). Flow cytometry using paraffin-embedded tissue: five years on. *Cytometry*, **10**, 229.

HELLMEN, E., LINGREN, A., LINELL, F., MATSSON, P. & NILSSON, A. (1988). Comparison of histology and clinical variables to DNA ploidy in canine mammary tumors. *Vet. Pathol.*, **25**, 219.

HIDDEMANN, W., SCHUMANN, J., ANDREEF, M. & 6 others (1984). Convention on nomenclature for DNA cytometry. *Cancer Genet. Cytogenet.*, **13**, 181.

JONKER, R., VAN ROTTERDAM, A., BAUMAN, J. & VISSER, J. (1987). Analysis of eight parameter listmode data and parameter estimation of uni- and bivariate distributions using a Kalman curve fitting procedure. *Cytometry* [suppl], **1**, 98.

KALLIONIEMI, O.-P., BLANCO, G., ALAVAIKKO, M. & 4 others (1987). Tumour DNA ploidy as an independent prognostic factor in breast cancer. *Br. J. Cancer*, **56**, 637.

MCGUIRE, W.L. & DRESSLER, L.G. (1985). Emerging impact of flow cytometry in predicting recurrence and survival in breast cancer patients. *J. Natl Cancer Inst.*, **75**, 405.

MINKE, J.M.H.M., CORNELISSE, C.J., STOLWIJK, J.A.M., KUIPERS-DIJKSHOORN, N.J., RUTTEMAN, G.R. & MISDORP, W. (1990). Flow cytometric DNA ploidy analysis of feline mammary tumors. *Cancer Res.*, **50**, 4003.

MORAN, R.E., BLACK, M.M., ALPERT, L. & STRAUS, M.J. (1984). Correlation of cell-cycle kinetics, hormone receptors, histopathology, and nodal status in human breast cancer. *Cancer*, **54**, 1586.

OLSZEWSKI, W., DARZYNKIEWICZ, Z., ROSEN, P.P., SCHWARTZ, M. & MELAMED, M.R. (1981). Flow cytometry of breast carcinoma: I. Relation of DNA ploidy level to histology and estrogen receptor. *Cancer*, **48**, 980.

RUSSO, J., GUSTERSON, B.A., ROGERS, A.E., RUSSO, I.H., WELLINGS, S.R. & VAN ZWIETEN, M.J. (1990). Biology of disease. Comparative study of human and rat mammary tumorigenesis. *Lab Invest.*, **62**, 244.

RUTTEMAN, G.R., CORNELISSE, C.J., DIJKSHOORN, N.J., POORTMAN, J. & MISDORP, W. (1988). Flow cytometric analysis of DNA ploidy in canine mammary tumors. *Cancer Res.*, **48**, 3411.

VAN ZWIETEN, M.J. (1984). *The Rat as Animal Model in Breast Cancer Research*. Martinus Nijhoff: The Hague, The Netherlands.

WILLIAMS, J.C., GUSTERSON, B., HUMPHREYS, J. & 4 others (1981). N-methyl-N-nitrosourea-induced rat mammary tumors. Hormone responsiveness but lack of spontaneous metastasis. *J. Natl Cancer Inst.*, **66**, 147.